# Chemoradiation in elderly esophageal cancer patients: rationale and design of a phase I/II multicenter study (OSAGE)

**DOI:** 10.1186/s12885-017-3465-4

**Published:** 2017-07-13

**Authors:** Stéphanie Servagi-Vernat, Gilles Créhange, Franck Bonnetain, Cécile Mertens, Etienne Brain, Jean François Bosset

**Affiliations:** 1 Department of radiotherapy, Institut de Cancérologie Jean Godinot, F-51100 Reims, France; 2 Departmentof radiotherapy, Centre Georges François Leclerc, F-21000 Dijon, France; 3 Methodology and Quality of life in Oncology Unbit, EA 3181, CHU Besançon, F-25000 Besançon, France; 4 Geriatric service, CHU Bordeaux, F-33000 Bordeaux, France; 5 Department of medical Oncology, CLCC Rene Huguenin Institut Curie, Saint Cloud, F-92210 Saint Cloud, France; 6 Department of radiotherapy, CHU Besançon, F-25000 Besançon, France

**Keywords:** Elderly patients, Esophageal cancer, Chemoradiotherapy, Quality of life, Clinical trial

## Abstract

**Background:**

The management of elderly patients with cancer is a therapeutic challenge and a public health problem. Definitive chemoradiotherapy (CRT) is an accepted standard treatment for patients with locally advanced esophageal cancer who cannot undergo surgery. However, there are few reports regarding tolerance to CRT in elderly patients. We previously reported results for CRT in patients aged ≥75 years. Following this first phase II trial, we propose to conduct a phase I/II study to evaluate the combination of carboplatin and paclitaxel, with concurrent RT in unresectable esophageal cancer patients aged 75 years or older.

**Methods/design:**

This prospective multicenter phase I/II study will include esophageal cancer in patients aged 75 years or older. Study procedures will consist to determinate the tolerated dose of chemotherapy (Carboplatin, paclitaxel) and of radiotherapy (41.4–45 and 50.4 Gy) in the phase I. Efficacy will be assessed using a co-primary endpoint encompassing health related quality of life and the progression-free survival in the phase II with the dose recommended of CRT in the phase I. This geriatric evaluation was defined by the French geriatric oncology group (GERICO).

**Discussion:**

This trial has been designed to assess the tolerated dose of CRT in selected patient aged 75 years or older.

**Trial registration:**

Clinicaltrials.gov ID: NCT02735057. Registered on 18 March 2016.

## Background

Cancer causes significant morbidity and mortality in the elderly and is an increasing healthcare issue. The French National Institute of Statistics and Economic Sciences (INSEE) estimates that about 200,000 centenarians will exist in France within 50 years [1]. Management of elderly cancer patients is therapeutically challenging and a public health problem. Chronological age does not always correlate with physiological organ impairment or poor performance status. Thus, in the elderly it is difficult to assess whether optimal treatment will be tolerated, making cancer management complex. Recently, significant progress has been made including development and validation of geriatric assessment tools with prognostic value to identify specific problems in the elderly cancer population [2–6]. Although age is not always related to performance status, older patients tend not to be considered for clinical studies. Thus, most patients aged 70 or older have traditionally been excluded from clinical studies. Since data concerning the management and outcome of elderly esophageal cancer patients are scarce, the optimal cancer management in this population remains uncertain. Esophagectomy, a standard treatment for early esophageal cancer in younger patients, is considered a high-risk surgery: with serious post-operative complications and in-hospital mortality rates between 1% to 23% [7, 8]. Finlayson et al. reviewed the esophagectomy outcomes of 27,957 patients ≥65 years old, using the Nationwide Inpatient Sample and Surveillance, Epidemiology and End Results-Medicare data, and showed that operative mortality significantly increased with age: 8.8% in patients 65–69 years, 13.4% in those 70–79 years, and 19.9% in those older than 80 years [9]. The most important objectives when managing elderly cancer patients are treatment duration, optimizing out-patient time, quality of life, and maintaining autonomy.

Definitive chemoradiotherapy (CRT) of 50 Gy over 5 weeks with either concomitant fluorouracil and cisplatin (4 cycles) or FOLFOX (6 cycles) is standard treatment for inoperable locally advanced esophageal cancer or for patients not considered as candidates for surgery; although this standard is not yet validated prospectively in patients older than 75 years [10–12]. However, data regarding tolerance to CRT in patients 75 years or older have been reported (Table 1).

**Table 1:** Summary of phase III and retrospective study of CRT for elderly patients with esophageal cancer

Authors	S	n	Age, mean, (range)	Treatment	Acute toxicities	Late toxicities	Survival
Song et al.	R	82	76 (70–87)	Paclitaxel 135 mg/m^2^ J1-J29	Leucopenia G4 10%	Esophageal stenosis 14,6%	mean PFS 18,2 m
				CDDP 30 mg/m^2^ J1 J3 - J29 J31	Esophagitis G4 2%	radiation pneumonia 7%	2 years PFS I-II 64%
				RTE 60 Gy	Thrombopenia G4 1%		2 years PFS III IV 21%
Su et al.	R	96	73 (65–82)	5FU		radiation pneumonia 15%	1 year PFS 70,9 m
				CDDP			3 years PFS 52 m
				RTE 56–66 Gy Extensive or conventional			
Zhong et al.	RA	79		RTE 56–59.4 Gy			mean PFS 19,7 m
				Docetaxel 25 mg/m^2^ and CDDP 25 mg/m^2^ weekly			1 year 78,5 / 61,2
				post RTCT Docetaxel 60 mg/m^2^ and CDDP 75 mg/m^2^			
Wang Jing et al.	R	100	76 (70–88)	CRT 50.4–66 CDDP-5FU-Docetaxel 4 courses	Leucopenia 21%	radiation pneumonia 13%	mean PFS CRT 15 m
					Esophagitis 12%		1 year PFS CRT 58%
					Pneumonia 10%		
Li et al.	R	32	74 (70–90)	RT 50–60 Gy	Esophagitis G3–4: 25%		mean PFS 23 m
				CT: Docetaxel weekly, CDDP-5FU, carboplatine-paclitaxel			
				paclitaxel only, doxifluridine			
Zhonghua et al.	R	89		RT 60 Gy: extensive or conventional	Leucopenia G3 33%		overall 3 year survival 32,8%
				CT paclitaxel 125 mg/m^2^ CDDP 20 (or oxaliplatine)			
Uno et al.	R	17	79 (75–85)	RT 50–60 Gy	Leucopenia G3 2%		median survival 9 months
				CT CDDP 5FU			OS 1 year 39%
Semrau et al.	R	15	74.1 (70–85)	RT 63 Gy		radiation pneumonia 4 pts	OS 13,9 m
				CDDP 20 mg/m^2^ and 5FU		Esophageal stenosis 9 pts	mean PFS 9,5 m
Anderson et al.	R	25	77 (66–88)	RT 50.4 Gy	Leucopenia G4 16%		mean OS 35 months
				CT 5FU Mitomycine			1 year OS 80%
Tougeron et al.	R	109	74.4 (70–88)	RTE 50–55 Gy	Any G3 22%		median OS 15,2 +/− 3 m
				5FU CDDP	one toxic death from sepsis		2 year survival rates 35,5%
Tougeron et al.	R	151	mean 75 +/−4.1	CRT 50–55 Gy, CDDP 5FU	Any G3 24.3% (mainly vomiting)		median OS 17,5 months
					and neutropenia		2-year survival 36,6%
Xu et al.	R	20	76 (70–88)	CRT 5FU CDDP	acute pneumonia G3–4: 5%		OS 17 months
							CRT mean PFS 14 months
Mak et al.	R	28	79.5 (75–89)	CRT 50.4 Gy 5FU CDDP	any G4 38%	Esophageal G3 17%	median survival 12.4 months
					any G3 73.5%	no late pulmonary	
					acute neutropenia G4: 23.5%		
					one death from sepsis		
Tumori et al.	R	57	69	CRT			median survival 11,2 months
Servagi et al.	RA	30	85 (79–92)	CRT 50Gy CDDP only or Oxalipatin only	Dysphagia G4 13.3%	radiation pneumonia 10%	PFS at 1 year 40%
							Three year OS 22%

Abbreviations: *S* study design, *R* retrospective study, *RA* Randomized study, *G* grade, *PFS* progression-free-survival, *CRT* chemoradiation, *n* number of patients, *CDDP* cisplatine

The phase III Dutch study randomly treated 368 patients, aged between 36 and 79 years, with resectable locally advanced esophageal cancer, to either CRT (180 patients): weekly carboplatin (AUC [area under curve] 2 mg/mL/min) and paclitaxel (50 mg/m^2^) combined with radiotherapy (RT): 41.4 Gy in 23 fractions, 5 days/week followed by surgery, or surgery alone (188 patients) [13]. In the CRT-surgery group, only 12/171 treated patients (7%) had grade 3 hematological toxicities; only 1 patient had a grade 4 hematologic toxicity and neutropenic fever. Furthermore, esophagitis grade ≥ 3 was uncommon (1%). A pathological complete response was observed in 47 patients (29%) in the CRT-surgery group [13]. The CRT-surgery group also had a significant better median OS 49.4 months, compared to 24.0 months in the surgery alone group (hazard ratio, 0.657; 95% confidence interval [CI], 0.495 to 0.871; *p* = 0.003).

We previously reported the results of a phase II single arm study evaluating platinum-based chemotherapy combined with RT (50 Gy) in patients 75 years or older with esophageal cancer [14]. Our data suggest that CRT, with acute toxicities, is feasible and tolerable in selected elderly patients with adequate functional status. However, the treatment efficacy was modest. Increase the RT dose or using new radiosensitizing agents may improve the therapeutic ratio or locoregional control. Noteworthy, half of the failures occurred within the irradiated volume.

We propose to conduct a phase I/II study to evaluate the combination of carboplatin and paclitaxel, with concurrent RT in unresectable esophageal cancer patients aged 75 years or older.

## Methods

### Objectives

Our multicenter phase I/II study aims to establish the optimal doses of RT associated with chemotherapy (carboplatin-paclitaxel) for elderly patients with inoperable esophageal cancer. The phase I will identify the maximum tolerated dose (MTD) of each component using 3 chemotherapy doses: 50%, 75%, and 100% of the standard dose established in the Dutch study (carboplatin: AUC 2 mg/mL/min; paclitaxel: 50 mg/m^2^; both administered weekly), and 3 RT doses: 41.4 Gy, 45 Gy, and 50.4 Gy. Each treatment component will be increased alternately. The MTD for carboplatin-paclitaxel will be defined according to the acute toxicity occurrence evaluated twice a week during treatment and once a week after the end of chemotherapy. Once the MTD of each component is established during the phase I, the recommended phase II dose (RP2D) will be defined. The phase II aims to assess the efficacy of the RP2D of RCT using the early tumor response rate at 8 weeks after treatment, confirmed at 12 weeks.

## Study objectives and evaluation criteria

The study protocol was approved by French national and regional ethics committees and the currently recruiting.

### Phase I

#### Primary objective

The phase I aims to determine the MTD and RP2D of concomitant RT and carboplatin-paclitaxel chemotherapy. The DLT for CRT is defined as the occurrence of any esophagitis grade ≥ 3 or any infection evaluated weekly during treatment and 1 month after treatment.

#### Secondary objectives

Secondary objectives are the compliance of the CRT, the acute toxicities evaluating with the CTCAE v4.03, the HR-QoL assess with validated instruments EORTC QLQ-C30 and ELD14 [15, 16], the progression-free-survival (PFS) and the overall survival (OS).

### Phase II

#### Primary objective

The phase II will assess the efficacy of CRT, using the tumor response rate (by RECIST) at 8 weeks, and confirmed at 12 weeks, after the end of treatment [17], but maintaining patients’ QoL.

#### Secondary objectives:

Secondary objectives will evaluate: CRT compliance, the acute and chronic toxicity (CTCAE v4.03), HR-QoL (EORTC QLQ-C30 and ELD14), PFS, OS, and the occurrence of radiation pneumonitis.

## Study population

### Eligibility criteria

Eligible patients must have a histology-confirmed esophageal squamous cell carcinoma or adenocarcinoma with tumors classified T1-T3, N0 N1, M1a (TNM 6th edition), with an Eastern Cooperative Oncology Group (ECOG) performance status (PS) ≤2, aged ≥75 years, neutrophil count ≥1.8.10^9^/L, platelet count ≥100.10^9^/L, hemoglobin ≥10 g/L, serum creatinine ≤1.25 μmol/L, and forced expiratory volume ≥ 1 L/s.

Patients who meet one of the following criteria will not be eligible: weight loss of ˃15% from normal weight, tumors classified T4 or M1b (TNM 6th edition), esophageal perforations or fistulas, previous chemotherapy or RT, mental retardation, and patients without written informed consent.

### Geriatric evaluation

Patients must meet the following criteria:

- Geriatric depression scale (GDS) <7/15
- Mini mental state examination (MMSE)/Folstein test >23/30
- Charlson comorbidity index ≤2 if ≥80 years old or ≤3 if 75 to 80 years old.
- Social support (at least one caregiver)
- No fall within the last 3 months
- Walking speed >0.8 m/s

This geriatric evaluation was defined by the French geriatric oncology group (GERICO). If a patient’s G8 score is ≤14/17 a geriatric intervention is recommended.

## Study procedures

### Treatments

#### Radiotherapy

RT is given concurrently on day (D)1 of the first chemotherapy cycle. The gross tumor volume (GTV) is the volume including the primary tumor and any involved lymph nodes. The clinical target volume (CTV) includes the GTV and a 3 cm craniocaudal margin around the primary tumor. The planning target volume (PTV) is the CTV with a 1 cm margin in all directions. No elective node RT is planned. The dose is prescribed according to the International Commission Radiation Units and Measurements (ICRU report 62 and 83). Conformal and intensity-modulated RT can be used in this study. The maximal dose to the spinal cord must be <44 Gy and 30% of the lung volume (volume of the two lungs minus the PTV) cannot receive ˃20 Gy (V_20_ ≤ 30%). For the heart, the volume of heart receiving at least 40 Gy must be <30% (V_40_ ≤ 30%). For the kidney (the sum of two kidneys), the volume receiving at least 18 Gy must be <50% (V_18_ < 50%). Cone beam computed tomography (CBCT) or kV-kV images to verify RT is required on the first three days of RT then once a week during RT. The RT doses and overall treatment duration is defined as follows:

*Phase I.*

41.4 Gy, 23 fractions, 1.8 Gy per fraction, over 4.6 weeks.

45 Gy, 25 fractions, 1.8 Gy per fraction, over 5 weeks.

50.4 Gy, 28 fractions, 1.8 Gy per fraction, over 5.6 weeks.

*Phase II.*

The RD2P established during the phase I.

Quality assurance.

The investigator will verify the treatment plan before initiating treatment. The following verifications are required for approbation: correct contouring of the GTV, CTV, and PTV; the dose homogeneity; and the respect of dose constraints.

#### Chemotherapy

The concurrent carboplatin-paclitaxel chemotherapy will be administered weekly in an outpatient unit. The chemotherapy dose will be according to the dose level assigned. According to the biological results, electrolytes will be compensated for and anemia will be corrected with erythropoietin or blood transfusion. Antiemetics (corticosteroids, 5-HT_3_ antagonists, and metoclopramide) will be used to prevent vomiting.

Phase I chemotherapy doses:

50% of the standard doses: carboplatin, AUC 1 and paclitaxel, 25 mg/m^2^.

75% of the standard doses: carboplatin, AUC 1.5 and paclitaxel, 37.5 mg/m^2^.

100% of the standard doses: carboplatin, AUC 2 and paclitaxel, 50 mg/m^2^.

Phase II: The recommended phase II doses of carboplatin and paclitaxel (RP2D).

**Fig. 1:**
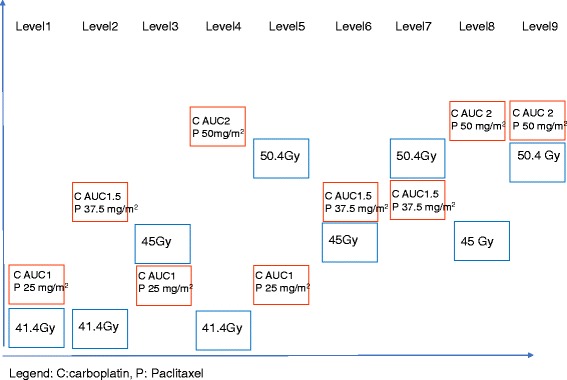
Description of the phase I CRT dose level. Abbreviations: CRT: Chemoradiation. C: Carboplatin. P: Paclitaxel

*Description of the phase I CRT dose levels* (Fig. 1).

Level 1.

### 41.4 Gy concomitant with 50% of the standard dose: Carboplatin, AUC 1 and paclitaxel, 25 mg/m^2^

Level 2.

41.4 Gy concomitant with 75% of the standard dose: carboplatin, AUC 1.5 and paclitaxel, 37.5 mg/m^2^.

Level 3.

## 45 Gy concomitant with 50% of the standard dose: Carboplatin, AUC 1 and paclitaxel, 25 mg/m^2^

Level 4.

41.4 Gy concomitant with 100% of the standard dose: carboplatin, AUC 2 and paclitaxel, 50 mg/m^2^.

Level 5.

### 50.4 Gy concomitant with 50% of the standard dose: Carboplatin, AUC 1 and paclitaxel, 25 mg/m^2^

Level 6.

45 Gy concomitant with 75% of the standard dose: carboplatin, AUC 1.5 and paclitaxel, 37.5 mg/m^2^.

Level 7.

50.4 Gy concomitant with 75% of the standard dose: carboplatin, AUC 1.5 and paclitaxel, 37.5 mg/m^2^.

Level 8.

45 Gy concomitant with 100% of the standard dose: carboplatin, AUC 2 and paclitaxel, 50 mg/m^2^.

Level 9.

50.4 Gy concomitant with 100% of the standard dose: carboplatin, AUC 2 and paclitaxel, 50 mg/m^2^.

## Study assessments

### Monitoring during treatment

Patients will be monitored weekly during CRT in the phase I and II, which will include:

- Clinical examination with intercurrent events, concomitant treatments, ECOG PS, weight, and dysphagia evaluation
- Hematology: white blood cell, neutrophils, hemoglobin, and platelets
- Biochemistry: aspartate aminotransferase (AST), alanine aminotransferase (ALT), bilirubin, creatinine, albumin, and pre-albumin
- Toxicity/symptoms: evaluation of treatment-related toxicities (CTCAE v4.03)
- Geriatric evaluation
- HR-QoL evaluations

### Assessments for the phase I

During and at 1 month after the treatment all esophagitis and infections must be declared.

### Post-treatment follow-up

Patients will be evaluated 1 month after the end of the CRT treatment, this evaluation will include: ECOG PS, weight, dysphagia evaluation, acute toxicities, blood cell count, ionogram, creatinine, AST, ALT, bilirubin, albumin, pre-albumin, and HR-QoL evaluations.

Imaging assessment (CT scan and esophagostomy) must be performed 8 weeks after the end of the treatment. To confirm a complete tumor response, a second imaging assessment must be performed 12 weeks after the end of the CRT treatment. To evaluate pulmonary effect, a thoracic radiography and a pulmonary function test will be performed 4 months after the end of the CRT treatment.

Following the initial 8-week post-treatment visit, patients will be assessed every 4 months until tumor progression. The evaluation will include: ECOG PS, weight, dysphagia, blood cell count, ionogram, creatinine, albumin, and pre-albumin, MMSE/Folstein test, completion of the EORTC QLQ C30 and ELD14. In addition, treatment-related toxicity/symptoms will be evaluated (CTCAE v4.03), as well as tumor assessment by imaging, if required.

## Study measurements

### Tumor response

Tumor will be assessed, by CT scan and esophagostomy, 8 weeks after the end of CRT treatment.

*CT scan: Esophageal tumor must be assessed by CT scan with the measure of the vertical length and maximal thickness on the transverse plane.

*Endoscopic complete response (CR) is defined as follows: disappearance of the tumor lesion, without stenosis, ulcerations, budding, and new lesion by endoscopy.

(All the endoscopic reports before and after treatment must be available for review, if required).

If the tumor assessment at 8 weeks is a complete response, a second CT scan and endoscopy must be performed at 12 weeks after treatment to confirm this result.

## Disease assessment by RECIST

**Complete response (CR)**: disappearance of all target lesions. Any pathological lymph nodes (whether target or non-target) must have reduced its shortest axis to <10 mm.

**Partial response (PR)**: A ≥ 30% decrease in the sum of diameters of target lesions, relative to the sum of diameters at baseline.

**Progressive Disease (PD)**: A ≥ 20% increase in the sum of diameters of target lesions, relative to the smallest sum of diameters during the study. In addition to the relative increase of 20%, the sum must also have an absolute increase of ≥5 mm. The appearance of one or more new lesions is also considered as a progression.

**Stable Disease (SD)**: The tumor shrinkage is not sufficient to qualify for PR nor has the tumor size increase sufficiently to qualify for PD relative to the smallest sum of diameters during the study.

### Time-related endpoints

*Event-free survival* is defined as the time from randomization until documented tumor progression or death, of any cause.

*Time to treatment failure* is defined as the time from randomization to treatment discontinuation for any reason: disease progression, treatment toxicity, patient’s refusal, patient lost to view, or death.

*Overall survival*: is measured from the time from randomization until death of any cause.

### Quality of life

HR-QoL in elderly cancer patients will be evaluated using EORTC QLQ-C30 and ELD14 [9].

The HR-QoL questionnaires will be completed by the patient before randomization and then at weekly study visit during CRT treatment, at 8 weeks after the end of CRT, and then every 4 months until tumor progression.

## Statistical considerations

The MTD is defined as the dose at which ˃20% of the patients experience a DLT. MTD identified in phase I will be the recommended phase II dose (RP2D). We plan to assess 9 dose levels, in phase I, with the following expected DLT rates for the CRT dose levels 1–9: 4%, 7%, 20%, 35%, 55%, 70%, 75%, 80%, and 85%.

We will use a continuous reassessment method (CRML) dose escalation design model as detailed in the methodology section [18].

Depending on the DLT observed within 30 days after induction phase of the previous patient and until first DLT occurrence, we will include 1 patient at dose level 1, 2 at dose level 2, 3 at the dose level 3 to 9. If DLT occurs the CRML will attribute to one patient the dose level for which the probability of toxicity was the closest of the achievable dose level (i.e. MTD).

We plan to include up to 24 patients in phase I. MTD/RP2D will be defined as the dose for which CRML will have attributed the dose level after the last included patients (i.e. the 24th patients) or if 9 patients have been treated at the same CRT dose level.

Once the MTD/RP2D, of carboplatin, paclitaxel, and RT, has been established patients will be included in phase II with the same eligibility criteria as the phase I. Overall 30 patients, phase I and II patients, will be treated at the MTD/RP2D. We expect a clinical CR of 50% evaluated at 8 weeks after treatment and confirmed by a clinical examination, esophagus transit, and CT scan at 16 weeks after treatment.

The data will be analyzed using a 3 steps Ensign Design with α = 5% (type I error) and 80% statistical power with the following hypotheses:

H0: clinical response (CR without decrease in QoL score) of 25% will be considered as uninteresting.

H1: clinical response of 50% is expected.

1st step: If we observe 1 clinical response, in the first 4 patients at the RP2D we will recruit 7 more patients.

2nd step: If we observe at least 3 clinical responses in the first 11 patients at the RP2D we will recruited 19 more patients.

3rd step: If we observe at least 12 clinical responses in the first 30 patients at the RP2D we will conclude that treatment is promising.

During phase I, at most 9 patients will be treated at any CRT dose level. The phase II will included up to 30 patients treated at the RP2D, these 30 will at most the 9 patients from phase I.

The maximum sample size for phase I and II is 54 patients. The statistical analysis plan will be finalized before the database is frozen. The main clinical and medical patients’ characteristics will be described for each dose level. Qualitative variables will be described using frequencies and percentages. Continuous variables (including QoL scores) will be described using means (SD) and medians (range). Distribution of continuous variables will be compared according to dose level with Wilcoxon tests.

DLT and tumor response rates will be described using frequencies and percentages with 95% confidence intervals (95% CIs). Similarly, toxicity grades and maximal toxicity (grade 3–4) will be reported at each follow-up evaluation and at each dose level. Response rates will be compared using Fisher exact test according to dose level. OS will be defined as the time from the study inclusion to death (of any causes). Surviving patients will be censored at the last follow-up. Median follow-up will be calculated according to reverse Kaplan-Meier estimates. OS curves will be plotted using the Kaplan-Meier method and described using medians with 95% CIs, and compared for exploratory purposes according to dose level using log-rank tests. QoL scores will be generated using EORTC algorithm guidelines and described at each follow-up for each dose level. The rate of missing items, scores, and questionnaires at each follow-up will be reported. Patients’ profiles will be generated according to missing QoL data patterns.

Clinical patients’ characteristics will be compared to these patients’ profiles to detect non-random missing data. Multiple imputations, taking into account the variables highlighted by the missing data study, will be done for sensibility analyses.

The following will be reported with 95% CI:

- A decrease in QoL scores ˃5 points relative to baseline at each follow-up and for each dose level,
- At least one decrease in QoL scores ˃5 points relative to baseline at each follow-up;
- At least one decrease QoL score ˃5 points relative to baseline will be reported during treatment.

Analyses of time until definitive deterioration of a QoL score (TUDD) will be estimated using Kaplan-Meier estimation for each dose level. The TUDD of score is defined as the time between inclusion and the first 5 points decrease in QoL score compared to baseline QoL score [19, 20]. Exploratory univariate and multivariate Cox model including time dependent covariates (time to DLT, time to first grade 3–4 toxicities, and time to progression) and other clinical variables will be performed to calculate hazard ratios with 95% CIs.

## Discussion

This multicenter prospective phase I/II protocol assesses CRT in patients aged 75 years or older with localized esophageal cancer.

Data are scarce regarding the use of CRT in localized esophageal cancer the principal studies are shown in Table 1. The literature, although based on small predominantly retrospective studies, provides evidence that elderly patients can tolerated and benefit from CRT.

The elderly are characterized by significant variability in aging; thus chronological age does not always reflect a patient’s ability to tolerate CRT. The comprehensive geriatric assessment, that aims to better evaluate the elderly, will be used to select this study population.

Esophageal cancer is associated with a poor prognosis, with a 5-year survival rate of 16% [21]. An objective of treatment in elderly cancer patients is to maintain QoL. In the phase II study a composite criterion associating tumor response with QoL was selected as the primary objective to account for the specific requirements for treating elderly cancer patients.
